# Human Mitochondrial RNA Processing and Modifications: Overview

**DOI:** 10.3390/ijms22157999

**Published:** 2021-07-27

**Authors:** Marta Jedynak-Slyvka, Agata Jabczynska, Roman J. Szczesny

**Affiliations:** Institute of Biochemistry and Biophysics, Polish Academy of Sciences, Pawińskiego 5A, 02-106 Warsaw, Poland; martaj@ibb.waw.pl (M.J.-S.); ajedroszkowiak@ibb.waw.pl (A.J.)

**Keywords:** mitochondria, mitochondrial transcription, RNA processing, RNA decay, RNA modifications, mitochondrial genome

## Abstract

Mitochondria, often referred to as the powerhouses of cells, are vital organelles that are present in almost all eukaryotic organisms, including humans. They are the key energy suppliers as the site of adenosine triphosphate production, and are involved in apoptosis, calcium homeostasis, and regulation of the innate immune response. Abnormalities occurring in mitochondria, such as mitochondrial DNA (mtDNA) mutations and disturbances at any stage of mitochondrial RNA (mtRNA) processing and translation, usually lead to severe mitochondrial diseases. A fundamental line of investigation is to understand the processes that occur in these organelles and their physiological consequences. Despite substantial progress that has been made in the field of mtRNA processing and its regulation, many unknowns and controversies remain. The present review discusses the current state of knowledge of RNA processing in human mitochondria and sheds some light on the unresolved issues.

## 1. Mitochondrial Genome Organization and Mitochondrial RNA Processing Compartmentalization

**Figure 1:**
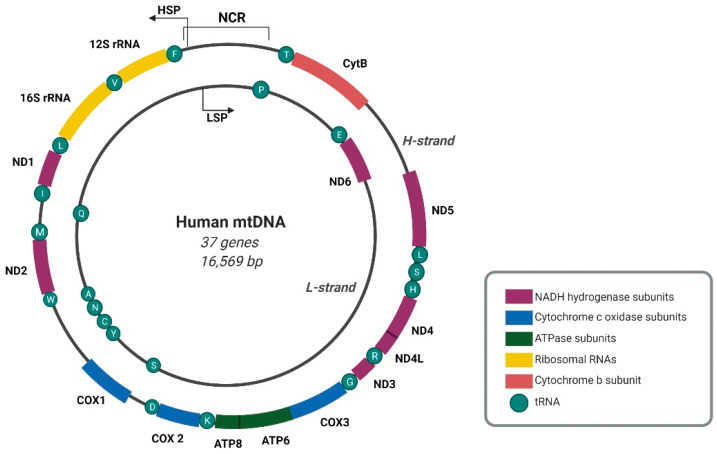
Map of the human mitochondrial genome. A double-stranded circular mitochondrial DNA (mtDNA) molecule includes 37 genes encoding 13 subunits of the OXPHOS system, 2 rRNAs, and 22 tRNAs. Transcription of both mtDNA strands initiates within the non-coding regulatory region (NCR), and the black arrows indicate transcription direction. HSP, LSP–transcription promoter of H- and L-strand, respectively. Open reading frames of *ATP8*/*ATP6* and *ND4L*/*ND4* are marked. Created with BioRender.com.

The human mitochondrial genome is a circular double-stranded DNA molecule of 16,569 base pairs, composed of heavy (H) and complementary light (L) strands that can be differentiated by the guanine (G) distribution [1,2]. Mitochondrial DNA (mtDNA) contains 37 genes that encode 13 subunits of the oxidative phosphorylation (OXPHOS) system, two ribosomal RNAs (rRNAs), and 22 transfer RNAs (tRNAs) [2] (Figure 1).

Mitochondrial genomes are packed into nucleoprotein complexes, referred to as mitochondrial nucleoids. The organized structure of nucleoids is maintained by various replication and transcription factors, one of which is mitochondrial transcription factor A (TFAM), which is known for its permanent association with mtDNA. To date, TFAM is the only protein with a well-established structural role in nucleoid organization. Based on the presence of two high-mobility group (HMG)-box domains, TFAM bends, wraps, and unwinds DNA, hence playing an important role in mtDNA packaging [3]. Among other nucleoid core factors, one can distinguish mtDNA polymerase γ (POLG), mitochondrial single-stranded DNA-binding protein (mtSSB), the NAD-dependent protein deacetylase sirtuin-1 (SIRT1), Twinkle helicase, and mitochondrial RNA polymerase (POLRMT) [4,5,6]. Replication and transcription are suggested to occur in the nucleoid core region, whereas RNA processing and translation are proposed to occur in the peripheral region that appears to be spatially coordinated with mitochondrial RNA granules (MRGs) and mitoribosomes [7,8]. Interestingly, some DNA-binding proteins, such as mtSSB and Twinkle, play a dual role in being associated not only with nucleoids and mtDNA, but also with MRGs, and their loss affects mitochondrial RNA (mtRNA) metabolism [9]. Although initial studies suggested multiple mtDNA copies per nucleoid [10], the application of high-resolution microscopy revealed only one or two mtDNA copies per nucleoid [11,12]. Nevertheless, the molecular basis of nucleoid division and the actual number of copies of mtDNA per nucleoid require further investigation.

**Figure 2:**
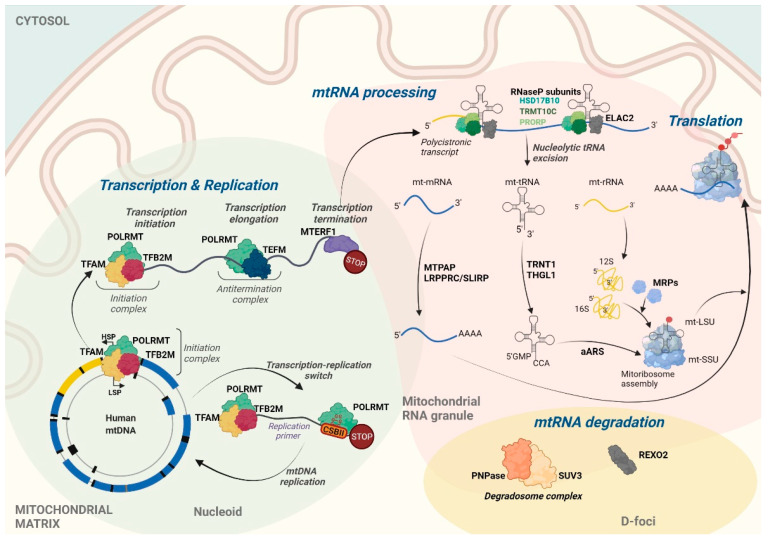
General overview of human mitochondrial gene expression. mtDNA replication and transcription occur within nucleoid structures (represented in light green) that spatially co-localize with mtRNA granules (MRGs, represented in light pink), which are suggested to be the place of post-transcriptional mtRNA processing and mitoribosome assembly. Degradation of mtRNA, mediated by the degradosome complex that involves SUV3 helicase and PNPase, occurs in D-foci that partially co-localize with MRGs. REXO2 exonuclease mediates the subsequent decay of RNA oligonucleotides. Aminoacyl-tRNA synthetases are abbreviated as aARS; mitochondrial ribosomal proteins are referred to as MRPs. Created with BioRender.com.

The transcription of both mtDNA strands is initiated within the major non-coding regulatory region (NCR). Almost all mitochondrial genes, including those that encode 12 subunits of the OXPHOS system, 2 mt-rRNAs (12*S* and 16*S*), and 14 mt-tRNAs, are transcribed from the template of the G-rich H-strand under control of the H-strand promoter (HSP). The complementary L-strand serves as a template for the production of only 1 mitochondrial messenger RNA (mt-mRNA) that encodes subunit 6 of NADH dehydrogenase (ND6), 8 mt-tRNAs, and mainly non-coding RNAs (ncRNAs) that are produced under control of the L-strand promoter (LSP) [2] (Figure 1). The transcription of both mtDNA strands spans nearly the entire length of mtDNA, resulting in the formation of long polycistronic transcripts (Figure 2). Primary mtRNAs undergo maturation and post-transcriptional modifications that are required for correct protein synthesis. The processing of mtRNAs is suggested to occur in ribonucleoprotein structures, referred to as MRGs [8,13]. In 2015, Antonicka and Shoubridge characterized the proteome of MRGs and identified a number of proteins that are associated with the granules. Among these proteins, several can be distinguished, including proteins that are involved in the maturation and processing of primary transcripts, proteins that are responsible for mtRNA degradation and turnover, mtRNA-modifying enzymes, structural proteins of the mitochondrial ribosome, and factors that are involved in ribosome assembly and disassembly [7] (Figure 2). Hence, MRGs were proposed to be centers of post-transcriptional mtRNA processing, the biogenesis of mitochondrial ribosomes, and the regulation of mitochondrial translation [7,14].

## 2. Transcription of the Human Mitochondrial Genome

Components of the basal apparatus that are required for the transcription of mtDNA include DNA-dependent POLRMT, TFAM, mitochondrial transcription factor B2 (TFB2M), and mitochondrial transcription elongation factor (TEFM) [15,16,17]. Structural studies have provided the precise mechanism of mitochondrial transcription initiation [17,18]. Transcription of the mitochondrial genome starts from the binding of TFAM to double-stranded mtDNA in a sequence-independent manner, with site specificity to the upstream −39 and −12 regions of the LSP and HSP, respectively, inducing mtDNA to bend into a U-turn shape. Following mtDNA binding, TFAM recruits POLRMT to the specific promoter via its N-terminal extension [18,19,20]. POLRMT is a single-subunit polymerase that contains two pentatricopeptide repeat (PPR) domains that are common for sequence-specific RNA-binding proteins (RBPs) [21]. The enzyme catalyzes the transcription of mtDNA into RNA and has high sequence homology with phage T7 and yeast mitochondrial RNA polymerases [22]. After the recruitment of POLRMT to the promoter by TFAM, the second factor, TFB2M, modifies the structure of POLRMT, which enables the opening of the promoter and the trapping of the non-template DNA strand [18,23].

In addition to TFAM, TFB2M, and TEFM, there are other factors are engaged in transcription regulation. One such example is mitochondrial ribosomal protein L7/L12 (MRPL12) that localizes to mitochondria in two distinct fractions, namely one fraction that is associated with mitoribosomes and another so-called “free” fraction. Wang et al. reported that MRPL12 interacts with POLRMT and stimulates polymerase activity in vitro, and that MRPL12 overexpression in vivo increases steady-state levels of mitochondrial transcripts [24]. The “free” fraction of MRPL12 interacts directly with POLRMT, thus selectively activating transcription and acting as a facilitator of the transition from the initiation to the elongation stage [25]. Another factor that is involved in regulation at the initiation stage is mitochondrial transcription rescue factor 1 (MTRES1). MTRES1 is an example of an RNA-binding protein that increases mitochondrial transcription upon stress conditions without affecting the stability of mitochondrial transcripts [26]. Kotrys et al. reported that MTRES1, which localizes to the mitochondrial matrix, interacts directly with POLRMT, whereas with TFAM, it associates in an RNA-mediated manner. The upregulation of MTRES1 that is observed upon the inhibition of mitochondrial transcription was proposed to be a rescue mechanism that protects against mtRNA loss under stress conditions [26].

The replication and transcription of human mtDNA are tightly coupled (Figure 2). TEFM serves as a molecular switch between these two processes [27]. Synthesis of the leading strand of mtDNA is initiated by the formation of an RNA primer. This primer is generated by the transcription of the L-strand that begins at the L-strand transcription start site and terminates ~100 bp downstream [28,29,30,31]. Termination occurs at the conserved G-rich sequence block II (CSBII), located in the vicinity of the replication origin [29]. Site-specific termination is caused by stable, four-stranded DNA-RNA G-quadruplex (G4) structures that are generated upon the transcription of G-rich CSBII [27,29]. In the elongation, full-length genome transcription mode, POLRMT requires the support of TEFM, which allows the polymerase to pass CSBII [27]. TEFM increases the processivity of elongation and stabilizes the elongation complex by binding to POLRMT, mtDNA, and the newly formed mtRNAs [18]. Molecular analyses have shown that TEFM binds POLRMT near the RNA exit channel, thereby preventing the formation of DNA-RNA G4 structures and the premature, replication-linked termination of L-strand transcription [18].

The precise mechanism of the termination of full-length mtDNA transcription is not fully understood. Mitochondrial transcription termination factor 1 (MTERF1), which binds to a 28-bp region immediately downstream of the 16*S* rRNA gene, was initially postulated to be responsible for the termination of the H-strand transcription at the 3′ end of 16*S* rRNA. However, it turned out that the protein terminates transcription from the opposite strand, preventing synthesis of antisense rRNA and transcription interference at the L-strand promoter [32]. The H-strand transcription termination factors remain unknown.

## 3. Maturation and Post-transcriptional Processing of RNA in Human Mitochondria

### 3.1. Processing of Primary Polycistronic Transcripts

The transcription of mtDNA leads to the formation of long polycistronic transcripts, including sequences of mRNAs, rRNAs, tRNAs, and ncRNAs. In human mitochondria, rRNA and the majority of mRNA coding sequences are separated by tRNA encoding genes. According to the “tRNA punctuation model”, mt-mRNAs and mt-rRNAs are released from the precursor transcript by the excision of mt-tRNAs (Figure 2) [33]. The exceptions are *MT-ND5*, *MT-CYB*, *MT-ATP8*/*6*, and *MT-CO3* mRNAs, which are not separated by tRNAs [2,33].

The canonical processing of polycistronic transcripts includes endonucleolytic cleavage at the 5′ and 3′ ends of mt-tRNAs, mediated by endoribonuclease P (RNase P) and elaC ribonuclease Z 2 (ELAC2, RNAseZ), respectively [34,35]. Mitochondrial RNase P, also known as a protein-only RNase P-like endoribonuclease complex, is composed of three proteins: tRNA methyltransferase 10C (TRMT10C, MRPP1), hydroxysteroid 17-β dehydrogenase 10 (HSD17B10, MRPP2), and protein-only RNase P catalytic subunit (PRORP, MRPP3) [34]. Mitochondrial RNase P does not contain the RNA component [34], in contrast to its nuclear counterpart [36]. In human cells, ELAC2 is present in two isoforms, a shorter form that localizes to the nucleus and its N-terminal extended form that contains the mitochondrial targeting sequence (MTS) that targets the protein to mitochondria [35,37].

Not all mitochondrial mRNAs are flanked by tRNAs and hence the processing of non-canonical cleavage sites of primary transcripts requires additional factors. The identity of all these factors is still unknown. However, members of the Fas-activated serine/threonine kinase (FASTK) protein family have been considered to be involved in this process. FASTK and its five homologs, FAST kinase domain-containing proteins 1-5 (FASTKD1-5), share the N-terminal MTS that ensures their mitochondrial localization and three conserved domains at the C-terminal end, namely FAST1, FAST2, and an RNA-binding domain abundant in apicomplexans (RAP) [38]. Structural and functional analyses have indicated that the RAP domain might be responsible for specific RNA binding. Homology predictions have also revealed that RAP folding resembles the PD-(D/E)-XK nuclease fold. For example, RAP domains from FASTKD1 and FASTKD4 were best fitted to the structure of very short patch repair (VSR) endonucleases [39]. Notably, D531 of the RAP domain (the residue that is highly conserved among various species) has been shown to be essential for the function of FASTKD4 and is homologous to the aspartate that is involved in the enzymatic activity of VSR endonuclease. Thus, the RAP domain has been suggested to possess endonucleolytic activity [39].

Each FASTK family member is involved in mitochondrial gene expression at different stages, from maturation and processing to mitoribosome assembly and translation. FASTK accumulates in MRGs, binding GRSF1 and MT-ND6 mRNA [40]. FASTK binds MT-ND6 mRNA at multiple sites within and downstream of the coding sequence in a RAP domain-dependent manner. The protein protects the MT-ND6 transcript from 3′-5′ degradation by the mitochondrial degradosome complex [40]. The depletion of FASTK results in the loss of MT-ND6 mRNA and decreases the activity of complex I [38]. FASTKD1 also localizes to MRGs and partially co-localizes with mtDNA. The enzyme negatively regulates MT-ND3 mRNA. Its loss results in the accumulation of MT-ND3 mRNA and increases the activity of complex I [39]. FASTKD2, similar to the above-mentioned kinases, localizes to MRGs [7] and associates with *MT-ND6* mRNA and 16*S* mt-rRNA [41]. The depletion of FASTKD2 results in the loss of 16*S* rRNA, which in turn disrupts mitoribosome assembly and translation [41]. Importantly, *FASTKD2* mutations are associated with severe human diseases. The pathogenic homozygous nonsense mutation causes cytochrome c oxidase deficiency, which is associated with infantile mitochondrial encephalomyopathy [42]. The heterozygous mutation leads to mitochondrial encephalomyopathy, lactic acidosis, and stroke-like (MELAS) syndrome [42,43]. Another member of the FASTK family, FASTKD3, localizes to the mitochondrial matrix but not to MRGs. This protein is required for the processing and maintenance of steady-state levels of *MT-ND2*, *MT-ND3*, *MT-CO2*, *MT-CYB*, and *MT-ATP8*/*6* mRNAs [44]. Moreover, it enables the efficient synthesis of COX1 protein and correct assembly of complex IV [44]. FASTKD4, similar to FASTKD3, localizes to the mitochondrial matrix but not to MRGs [39]. It binds to the majority of H-strand-derived transcripts and regulates the stability of *MT-ATP8*/*6*, *MT-CO1*, *MT-CO2*, *MT-CO3*, *MT-ND3*, *MT-CYB*, and *MT-ND5* mRNAs [39].

The last member of the family, FASTKD5, localizes to MRGs, where it regulates almost all mt-mRNAs, and is responsible for maintaining proper protein synthesis [7]. Antonicka and Shoubridge reported that FASTKD5 is involved in the non-canonical processing of primary transcripts, in which depletion of the protein led to the substantial accumulation of H-strand unprocessed precursor mRNAs, which in turn resulted in the loss of the mitochondrial small subunit (mt-SSU) and large subunit (mt-LSU), and the disassembly of the mitoribosome [7]. It remains unknown whether FASTKD5 has nucleolytic activity and processes these transcripts itself or if it requires the support of other factors. Notable is NLRX1, a nuclear-encoded protein that translocates to the mitochondrial matrix. Singh et al. reported that NLRX1, a member of the nucleotide-binding oligomerization domain-like receptor (NLR) family, localizes to MRGs and specifically interacts with FASTKD5 [45]. These authors showed that the loss of NLRX1 significantly increased *MT-ND5*, *MT-CO1-3*, *MT-ATP8*/*6*, and *MT-CYB* mRNA levels, whereas NLRX1 overexpression decreased the levels of 16*S* rRNA and most mature mt-mRNAs, with the exception of *MT-ND2*, *MT-ND4L*/*ND4*, *MT-ND3*, and *MT-ND6* [45]. Moreover, the ectopic expression of NLRX1 decreases OXPHOS activity and assembly, and affects the organization of mitochondrial supercomplexes, indicating its importance for mitochondria biogenesis [45].

### 3.2. Polyadenylation and Aminoacetylation

After excision from polycistronic transcripts, mt-mRNAs undergo maturation. One of the most prevalent post-transcriptional modifications of mt-mRNAs is polyadenylation of the 3′ end, and almost all mt-mRNAs, except *MT-ND6*, are subjected to this process [46]. Polyadenylation is performed by mitochondrial poly(A) polymerase (MTPAP) [47,48]. Following import into mitochondria, MTPAP localizes to MRGs [7,49]. The post-transcriptional addition of adenines to the 3′ end is essential for mt-mRNAs that lack the complete stop codon. After endoribonucleolytic processing, they carry only the U or UA at the 3′ end. One function of MTPAP is to complete the missing stop codons (UAA) and thus the open reading frame [50].

MTPAP is also important for the maturation and repair of mt-tRNA [51]. In human mitochondria, tRNA_Tyr_ and tRNA_Cys_ encoding genes overlap by one nucleotide, and their processing results in the release of tRNA_Tyr_ that lacks the adenosine at the 3′ end. MTPAP introduces the discriminator adenosine to mt-tRNA_Tyr_, which is essential for subsequent aminoacylation [51]. The discriminator base is located at position 73 of tRNAs upstream of the added CCA sequence, and is strongly preferred by aminoacyl-tRNA synthetases and tRNA nucleotidyltransferases [52]. Notably, MTPAP is unable to add a single adenosine to the 3′ end of mt-tRNAs. Therefore, following adenylation, mt-tRNA_Tyr_ requires the trimming of oligo(A) tails to a single nucleotide. Based on in vitro studies, it has been proposed that oligo(A)-trimming may be performed either by ELAC2 and/or 3′ exonuclease phosphodiesterase 12 (PDE12) that cleaves the 3′,5′-phosphodiester bond within oligoadenylates [51,53]. In human cells, PDE12 is required for the removal of the aberrant adenylation of mitochondrial 16*S* rRNA and tRNAs, which allows for efficient aminoacylation, thereby ensuring proper maturation and translation [53]. However, the absence of PDE12 does not affect mt-mRNA poly(A) tail length or stability [53]. Additionally, the polyadenylation of structurally abnormal mt-tRNAs has been proposed to trigger their degradation [54]. Toompuu et al. showed that mt-tRNAs undergo polyadenylation, followed by rapid degradation, upon treatment with high concentrations of ethidium bromide, which suppresses mitochondrial transcription and, at high doses, efficiently intercalates to mt-tRNAs and potentially changes their structure [54].

Interestingly, mt-rRNAs and non-coding RNAs can also be polyadenylated [53,55,56]. The role of this process is not fully understood. However, one possibility is that, similar to the modification of abnormal mt-tRNAs, the polyadenylation of mitochondrial antisense transcripts stimulates their decay. Dysfunction of the RNA helicase SUV3, a component of mitochondrial RNA decay machinery, results in the upregulation of mtRNA molecules with extended poly(A) tails [56]. This would be a hallmark of the bacterial origin of mitochondria, in which polyadenylation marks RNAs for degradation [55,57]. The fact that stable, mature mt-mRNAs have poly(A) tails suggests that the adenylation of mitochondrial transcripts may have different functions, depending on the molecular context. In the nucleus, the impact of polyadenylation on RNA stability, export, processing, and decay depends on the molecular machinery that catalyzes the modification [58]. MTPAP is suggested to be the sole 3′ end mtRNA polyadenylation enzyme, at least in *Drosophila melanogaster* [59]. Nevertheless, this modification appears to exert differential effects on mtRNAs. Notably, the length of A-tails in specific mt-mRNAs is heterogeneous and varies both within the same cell type and within the same transcript between different cell types [60]. Two fractions are generally observed: poly- and oligoadenylated mt-mRNAs. Some transcripts have two longer poly(A) tail extensions, such as *MT-CO1* and *MT-CO3*, which have median lengths of 37 and 52 nt or 42 and 57 nt, respectively. *MT-ND5* is the most divergent from other mitochondrial transcripts. In *MT-ND5*, although longer tails (25 or 35 nt) may be observed, a significant fraction is oligoadenylated, with an A-tail extension length of <10 nt, wherein a non-adenylated population occurs most frequently [60]. Altogether, polyadenylation is not only required for mt-mRNA translatability, but may also play a role in mtRNA surveillance. Consequently, perturbation of the polyadenylation of mtRNAs affects mitochondrial translation [51,61].

Another modification that plays a crucial role in mtRNA post-transcriptional regulation is the aminoacylation of tRNAs, i.e., the attachment of an amino acid to a tRNA. Mitochondrial tRNAs must undergo aminoacylation to deliver amino acids for translation. Enzymes that are responsible for this process include mitochondrial aminoacyl-tRNA synthetases (aaRSs). Human mitochondria contain 19 aaRSs. All members of this family possess a tRNA anticodon-binding domain and a catalytic domain. The majority of mitochondrial aaRSs are encoded by genes that are different from their cytoplasmic counterparts. Exceptions include mitochondrial LysRS and GlyRS, which are produced as a result of alternative splicing or an alternative translation initiation site, respectively [62,63,64]. Each aaRS catalyzes mt-tRNA charging with a cognate amino acid, with the exception of mt-tRNA_Gln_. To date, no gene that encodes mt-GlnRS has been discovered. Instead, Nagao et al. have shown that in human mitochondria, Gln-charging of mt-tRNA_Gln_ occurs via an indirect two-step pathway. First, non-discriminating GluRS catalyzes the misaminoacylation of tRNA_Gln_ with glutamic acid, resulting in the formation of Glu-tRNA_Gln_. Subsequently, the glutamate residue is transamidated by the glutamyl-tRNA_Gln_ amidotransferase hGatCAB to obtain Gln-tRNA_Gln_ [65].

### 3.3. Post-transcriptional Chemical Modifications of mRNAs

Chemical modifications that occur post-transcriptionally are crucial for extending the properties of the four basic nucleotides. Thus, they are involved in the biogenesis, stability, and function of all mtRNA species. Post-transcriptional modifications of mt-mRNAs that have been discovered to date cover methylation and pseudouridylation (Table 1). Two tRNA methyltransferase complexes, TRMT6/61A and TRMT61B, known for the methylation of mt-tRNAs, are also responsible for the *N*^1^-methyladenosine (m^1^A) modification of mt-mRNAs [66,67]. There are 22 TRMT6/61A-dependent m^1^A sites in 10 of 13 mt-mRNAs, the majority of which are located in the coding region (CDS), with only one in the 5′-untranslated region (UTR) [67]. TRMT61B methyltransferase catalyzes the m^1^A methylation of *MT-CO2* and *MT-CO3* mRNAs, and its depletion correlates with lower levels of m^1^A modifications [66,67]. The m^1^A methylation of mt-mRNAs within the CDS blocks canonical A:U base pairing and prevents protein synthesis because effective translation requires accurate base pairing between mRNA codons and cognate tRNAs anticodons [67]. Additionally, adenosine at the N^1^ position is methylated in *MT*-*ND5* mRNA by TRMT10C, the RNase P complex subunit [66].

Pseudouridine synthases are enzymes that are responsible for pseudouridylation by converting specific uridines to pseudouridines (Ψ). The mitochondrial mRNA pseudouridine synthases RPUSD3 and TRUB2 introduce Ψ at position 391 of *MT-CO1* and positions 698–700 of *MT-CO3* mRNAs [68]. RPUSD3 is postulated to be the major enzyme that is responsible for the pseudouridylation of mt-mRNAs, whereas TRUB2 appears to play a secondary role [68]. The functional consequences of mt-mRNA pseudouridylation have not been explored yet.

### 3.4. Post-transcriptional Chemical Modifications of tRNAs

**Figure 3:**
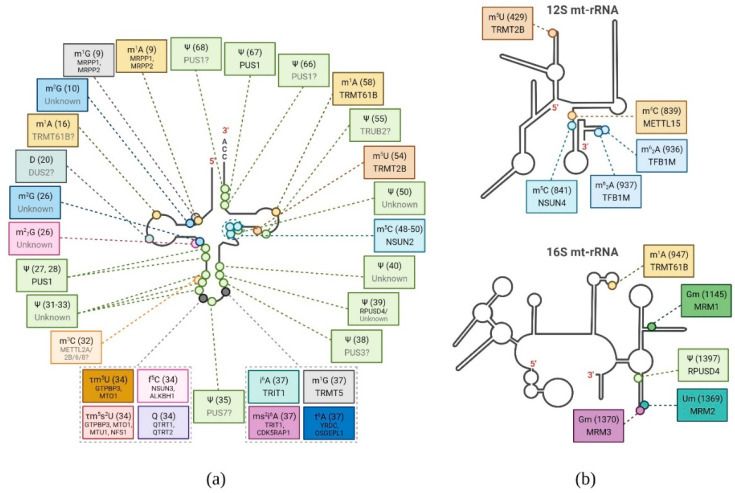
Summary of post-transcriptional base modification of human mt-tRNAs and mt-rRNAs. Schematic representation of the secondary structure of: (**a**) generic mt-tRNA; and (**b**) 12*S* and 16*S* mt-rRNA with indicated post-transcriptional base modifications (circles) identified in humans. Chemical modification, base position number (in brackets), and enzyme responsible (if known) for each position are depicted in boxes. The modifying enzymes predicted to be responsible for the indicated modification, but not experimentally confirmed, are indicated in gray and followed by a question mark. Modifications shown: 1-methyladenosine (m^1^A), 1-methylguanosine (m^1^G), *N*^2^-methylguanosine (m^2^G), dihydrouridine (D), *N*^2^,*N*^2^-dimethylguanosine (m^2^_2_G), pseudouridine (Ψ), 3-methylcytidine (m^3^C), 5-taurinomethyluridine (τm^5^U), 5-taurinomethyl-2-thiouridine (τm^5^s^2^U), 5-formylcytidine (f^5^C), Queuosine (Q), N^6^-isopentenyladenosine (i^6^A), 2-methylthio-*N*^6^-isopentenyladenosine (ms^2^i^6^A), *N*^6^-threonylcarbamoyladenosine (t^6^A), 5-methylcytidine (m^5^C), 5-methyluridine (m^5^U), *N*^4^-methylcytidine (m^4^C), *N*^6^,*N*^6^-dimethyladenosine (m^6^_2_A), 2′-*O*-methylguanosine (Gm), and 2′-*O*-methyluridine (Um). Created with BioRender.com.

Mitochondrial tRNAs undergo extensive post-transcriptional modifications (Figure 3 and Table 1). At position 9 of mt-tRNAs, adenine or guanine is methylated to N^1^-methyladenine (m^1^A9) and *N*^1^-methylguanine (m^1^G9), respectively. The MRPP1/MRPP2 subcomplex, composed of two subunits of RNase P, is responsible for both m^1^A9 and m^1^G9 methylation [69]. A relatively abundant modification, m^2^G10, is found in 15 mt-tRNA species, whereas the m^1^A modification is present exclusively at position 16 in mt-tRNA_Arg_. TRMT61B is predicted to be responsible for the specific m^1^A16 modification [70].

One key modified mt-tRNA position is position 34 located within the anticodon sequence, also named the “wobble position”. Different nucleoside modifications observed in this position, such as 5-formylcytosine and queuosine, improve codon recognition selectivity. Two enzymes are responsible for the formation of 5-formylcytosine (f^5^C) at position 34 of the anticodon in mt-tRNA_Met_. The first enzyme, the tRNA (cytosine(34)-C(5))-methyltransferase (NSUN3), methylates the cytosine to 5-methylcytosine (m^5^C) [71]. The second enzyme, AlkB homologue 1 (ALKBH1), catalyzes the formation of f^5^C at this position [72]. Both NSUN3 and ALKBH1 modify position 34 of mt-tRNA_Met_ to enable the recognition of alternative codons that encode methionine [71,73]. Interestingly, elevated levels of m^1^A16 in mt-tRNA_Arg_ and m^1^A58 in mt-tRNA_Lys_ were observed in ALKBH1 knockout cells, suggesting that ALKBH1 may also have demethylation activity toward m^1^A in some mt-tRNAs [72]. The tRNA modification GTP-binding protein 3 (GTPBP3) and the protein MTO1 homolog (MTO1) together catalyze the taurinomethylation of uridine at the wobble position (τm^5^U34) in mt-tRNA_Lys_, mt-tRNA_Glu_, mt-tRNA_Gln_, mt-tRNA_Leu_^UUR^, and mt-tRNA_Trp_ [74,75]. Another enzyme, mitochondrial tRNA-specific 2-thiouridylase (MTU1, TRMU), introduces the additional 2-thiolation of 5-taurinomethylridine (τm^5^s^2^U34) at the wobble position in mt-tRNA_Lys_, mt-tRNA_Glu_, and mt-tRNA_Gln_ [74,76]. Queuosine (Q) is also present at position 34 of four mt-tRNA species (tRNA_Tyr_, tRNA_His_, tRNA_Asp_, and tRNA_Asn_). tRNA guanine transglycosylase (TGT), composed of two subunits (catalytic QTRT1 and non-catalytic QTRT2), is responsible for the substitution of guanine with queuosine in cytoplasmic tRNAs. It is also essential for the modification of mt-tRNAs [70]. Suzuki et al. reported that Q34 in mt-tRNA_Tyr_ is important for the efficient decoding of the UAU codon [70].

Another site is position 37 downstream of the anticodon that also needs to be modified to stabilize codon–anticodon interactions. The enzymes that are responsible for modifying mt-tRNA at position 37 include tRNA dimethylallyltransferase (TRIT1) and tRNA (guanine(37)-N1)-methyltransferase (TRMT5). TRIT1 introduces the dimethylallyl group to the adenine, resulting in the formation of *N*^6^-(dimethylallyl)adenosine (i^6^A37) in mt-tRNA_Phe_, mt-tRNA_SerUCN_, mt-tRNA_Trp_, and mt-tRNA_Tyr_ [70,77], whereas TRMT5 specifically methylates the N^1^ position of the guanosine (m^1^G37) in mt-tRNA_Gln_, mt-tRNA_LeyCUN_, mt-tRNA_Trp_, and mt-tRNA_Tyr_ [78]. In turn, mt-tRNAs with i^6^A37 are subsequently modified by cyclin-dependent kinase 5 regulatory subunit associated protein 1 (CDK5RAP1) that catalyzes 2-methylothiolation (ms^2^i^6^A37) [79]. Another modification at position 37 is N6-threonylcarbamoyladenosine (t^6^A), which is essential for translation accuracy and fidelity. Two enzymes are responsible for this modification in human mitochondria. First, YRDC synthesizes an L-threonylcarbamoyl adenylate (TC-AMP) intermediate, and then the TC moiety is transferred to five mt-tRNAs (tRNA_Ile_, tRNA_Lys_, tRNA_Asn_, tRNA_Ser(AGY)_, and tRNA_Thr_) by probable tRNA N6-adenosine threonylcarbamoyltransferase, OSGEPL1 [70,80,81].

5-Methylcytidine (m^5^C) has been identified in six human mt-tRNAs at position 48 (tRNA_Phe_, tRNA_His_, tRNA_Leu(UUR)_, tRNA_Tyr_, and tRNA_Ser(AGY)_), position 49 (tRNA_Glu_ and tRNA_Ser(AGY)_), and position 50 (tRNA_Ser(AGY)_). The NOP2/Sun RNA methyltransferase family member 2 (NSUN2) protein is necessary for this modification [82,83]. 5-Methyluridine (m^5^U) has also been identified at position 54, which is unique to mitochondrial tRNA_Pro_, tRNA_Asn_, tRNA_Leu(UUR)_, tRNA_Ser(UCN)_, and tRNA_Gln_ and catalyzed by TRMT2B [84]. Human mt-tRNAs, such as tRNA_Leu(UUR)_, tRNA_Lys_, tRNA_Ser(UCN)_, tRNA_Cys_, tRNA_Glu_, and tRNA_Ile_, may also be methylated (m^1^A) at position 58 by mitochondria-specific TRMT61B [85].

Another commonly occurring mt-tRNA modification is pseudouridylation, known for the stabilization of tRNA stacking [86]. Pseudouridine synthetase 1 (PUS1) modifies U27 and U28 in the anticodon of mt-tRNAs, whereas RNA pseudouridylate synthase domain-containing protein 4 (RPUSD4) introduces Ψ at position 39 of mt-tRNA_Phe_ [68,87,88]. Combining mass spectrometry analyses, the biochemical mapping of Ψ in mt-tRNAs, and previously published data, Suzuki et al. indicated 52 Ψ sites in all species of human mt-tRNAs. Of these, 44 were confirmed by tRNA-Ψ-sequencing [70].

Importantly, mt-tRNAs may also be modified at their 5′ and 3′ ends. The enzyme tRNA nucleotidyl transferase 1 (TRNT1) is responsible for the addition of the CCA sequence at the 3′ end of mt-tRNAs [89], whereas tRNA_His_ guanylyltransferase (THG1L) introduces the guanosine monophosphate moiety to the 5′ end of mt-tRNA_His_ after transcription and RNase P processing, which is an essential step for proper translation [90].

### 3.5. Post-transcriptional Chemical Modifications of rRNAs

Human mitoribosomes consist of 12*S* (small subunit) and 16*S* (large subunit) rRNAs and 82 mitoribosomal proteins (MRPs) that are encoded in the cell nucleus and imported to mitochondria. Only three types of modifications (2′-*O*-methylation, nucleobase methylation, and pseudouridylation) and 10 modified sites have been hitherto identified in human mt-rRNAs (Figure 3b and Table 1).

16*S* rRNA undergoes 2-*O*-ribose methylation at three sites (Gm1145, Um1369, and Gm1370), catalyzed by mitochondrial rRNA methyltransferase 1 (MRM1), MRM2, and MRM3, respectively [91,92,93]. The latter two modified sites occur within the 16*S* rRNA A-loop, which is an essential component of the peptidyl transferase center. The absence of these modifications disrupts the assembly of the mitochondrial ribosome large subunit and thus mitochondrial translation [92]. Among modifications of 16*S* rRNA, one can also distinguish m^1^A base methylation at position 947, catalyzed by TRMT61B [94], and pseudouridylation at position 1397, mediated by RPUSD4, which are essential for transcript stability, mt-LSU assembly, and mitochondrial translation [68,88].

12*S* rRNA undergoes m^6^_2_A dimethylation at positions 936 and 937, catalyzed by TFB1M, a paralogue of the mitochondrial transcription factor TFB2M [95]. Adenosine dimethylation in 12*S* rRNA is necessary for the binding of the ribosome-binding factor A (RBFA) and the proper assembly of mt-SSU [96,97,98]. 12*S* rRNA also undergoes methylation in several positions. The methylation of cytosine at position 841 (m^5^C841) is catalyzed by NSUN4 [99]. The m^5^C841 modification is likely to be important for mitoribosome biogenesis. It has been shown that NSUN4 knockout in mice abolishes translation [99]. The m^4^C methylation at position 839 is introduced by methyltransferase METTL15 [100], which is essential for proper translation as the depletion of the METTL15 protein impairs mitoribosome assembly [101]. TRMT2B methyltransferase that catalyzes uridine methylation in tRNA is also responsible for methylation at position 429 (m^5^U429) in 12*S* rRNA [102]. The contribution of TRMT2B to mitochondrial translation requires further investigation as human cells with TRMT2B knockout did not display the phenotype with regards to RNA stability, mitochondrial translation, or cellular growth [102].

**Table 1:** Post-transcriptional modifications of mtRNAs in human mitochondria. f^5^C: 5-Formylcytosine; m^1^A: 1-ethyladenosine; m^1^G: 1-Methylguanosine; m^2^G: *N*^2^-Methylguanosine; m^2^_2_G: *N*^2^,*N*^2^-Dimethylguanosine; m^3^C: 3-Methylcytosine; m^4^C: *N*^4^-Methylcytosine; m^5^C: 5-Methylcytosine; m^6^_2_A: *N*^6^,*N*^6^-Dimethyladenosine; ms^2^i^6^: 2-Methylthio-*N*^6^-isopentenyladenosine; t^6^A*: N*^6^-Threonylcarbamoyladenosine; Q: 7-Deazaguanosine (queuosine); Ψ: pseudouridine.

Transcript	Position	Modification	Modifying Enzyme	Ref.
**mRNA**				
*MT-CO1*	1472	m^1^A	TRMT6/61A, TRMT61B	[66,67]
	391	Ψ	RPUSD3, TRUB2	[68]
*MT-CO2*	297	m^1^A	TRMT6/61A, TRMT61B	[66,67]
*MT-CO3*	707	m^1^A	TRMT6/61A, TRMT61B	[66,67]
	698–700	Ψ	RPUSD3, TRUB2	[68]
*MT-ND5*	1374	m^1^A	TRMT10C	[66]
**tRNA**				
Asn, Arg, Asp, Ala, His, Gly Glu, Leu(CUN), Lys, Pro, Phe, Val, Trp, Thr	9	m^1^A	MRPP1/MRPP2	[69]
Gln, Cys, Ile, Leu(UUR), Tyr		m^1^G	MRPP1/MRPP2	[69]
Ala, Asp, Glu, Phe, Gly, His, Lys, Leu(UUR), Leu(CUN), Tyr, Trp, Val, Asn, Thr, Val	10	m^2^G	Unknown	[70]
Arg	16	m^1^A	Predicted: TRMT61B	[70]
Leu(UUR), Asn, Gln	20	D	Predicted: DUS2	[70,103]
Ala, Glu, Arg	26	m^2^G	Unknown	[70]
Ile		m^2^_2_G	Unknown	[70]
Asn, Asp, Cys, His, Ile, Leu(UUR), Leu(CUN), Met, Pro, Val, Ser(UCN), Tyr, Lys	27	Ψ	PUS1	[70,87]
Asn, Cys, Ala, Leu(CUN), Ser(UCN), Lys, Glu, Tyr, Phe, Gly, Ile	28	Ψ	PUS1	[70,87]
Leu(CUN)	31	Ψ	Unknown	[70]
Cys, Pro, Arg	32	Ψ	Unknown	[70]
Ser(UCN), Thr		m^3^C	Predicted METTL2A, METTL2B, METTL6, or METTL8	[70]
Gln	33	Ψ	Unknown	[70]
Lys, Glu, Gln, Leu(UUR), Trp	wobble position 34	τm^5^U	GTPBP3, MTO1	[74]
Lys, Glu, Gln		τm^5^s^2^U	GTPBP3, MTO1, MTU1, NFS1	[74,76]
Met		f^5^C	NSUN3, ALKBH1	[71,72]
Asp, His, Asn, and Tyr		Q	QTRT1, QTRT2	[70]
His	35	Ψ	Predicted: PUS7	[70]
Phe, Ser(UCN), Trp, Tyr, Cys	37	i^6^A	TRIT1	[70,77]
Phe, Ser(UCN), Trp, Tyr		ms^2^i^6^A	TRIT1, CDK5RAP1	[79]
Gln, Leu(CUN), Pro, Ala		m^1^G	TRMT5	[78]
Ser(AGY), Thr, Asn, Ile, Lys		t^6^A	YRDC, OSGEPL1	[70,80,81]
Ala, Pro	38	Ψ	Predicted: PUS3	[70]
Ala, Pro, Cys, Gly, His, Gln, Arg, Val, Tyr	39	Ψ	Unknown	[70]
Phe			RPUSD4	[88]
Glu, Gly Asn, Gln	40	Ψ	Unknown	[70]
Phe, His Leu(UUR), Ser(AGY), Tyr	48	m^5^C	NSUN2	[70,82,83]
Glu, Ser(AGY)	49	m^5^C	NSUN2	[70,82,83]
Ser(AGY)	50	m^5^C	NSUN2	[70,82,83]
Met		Ψ	Unknown	[70]
Pro, Asn, Leu(UUR), Ser(UCN), Gln	54	m^5^U	TRMT2B	[84,102]
Glu, Met, Leu(UUR), Ser(UCN), Asn, Pro, Gln	55	Ψ	Predicted: TRUB2	[70,104]
Leu(UUR), Lys, Ser(UCN) Cys, Glu, Ile	58	m^1^A	TRMT61B	[85]
Pro	66	Ψ	Predicted: PUS1	[70]
Pro	67	Ψ	PUS1	[70]
Ala	68	Ψ	Predicted: PUS1	[70]
**rRNA**				
12*S*	429	m^5^U	TRMT2B	[102]
	839	m^4^C	METTL15	[100]
	841	m^5^C	NSUN4	[99]
	936	m^6^_2_A	TFB1M	[95,96,97]
	937	m^6^_2_A	TFB1M	[95,96,97]
16*S*	947	m^1^A	TRMT61B	[91]
	1145	Gm	MRM1	[93]
	1369	Um	MRM2	[92]
	1370	Gm	MRM3	[92]
	1397	Ψ	RPUSD4	[68]

## 4. Mitochondrial RNA Surveillance and Decay

Similar to other genetic systems, steady-state levels of mtDNA-encoded transcripts are an outcome of their synthesis and decay, which depend on RNA transcription, degradation, and stabilization factors. One RNA-binding protein that is engaged in maintaining the stability, and preventing the degradation, of mtRNAs is the leucine-rich pentatricopeptide repeat (PPR)-containing protein (LRPPRC). LRPPRC localizes to the mitochondrial matrix. Following its import into mitochondria, LRPPRC associates with the stem-loop-interacting RNA-binding protein (SLIRP), forming the LRPPRC/SLIRP complex. This complex binds predominantly mt-mRNAs and mt-rRNAs [105]. LRPPRC is the factor that is responsible for RNA binding, whereas SLIRP alone does not bind RNA or affect mt-mRNA polyadenylation [106,107,108]. Although SLIRP contains the RNA recognition motif (RRM) domain, this domain is responsible for the protein–protein interactions required for the formation of the LRPPRC/SLIRP complex, but is not responsible for RNA binding [108]. Structural studies have shown that the LRPPRC-SLIRP complex is associated with translating mitoribosomes via PPR-mediated interactions with the mitoribosomal PPR protein mS39 and delivers mt-mRNAs to the mitoribosomal small subunit [109]. The presence of LRPPRC in mitochondria was positively correlated with mt-mRNA polyadenylation [106]. The loss of LRPPRC results in the shortening of poly(A) tails, a decrease in the stability of HSP-derived transcripts, and misregulated translation [105,106]. Additionally, the LRPPRC/SLIRP complex suppresses PNPase-mediated mt-mRNA decay [110].

Recent work by Bruni et al. suggests that the stability of mt-mRNAs also depends on mitoribosome binding [111]. Depletion of the mt-LSU results in the downregulation of mt-mRNA levels, whereas the depletion of both mt-LSU and mt-SSU partially recovers mtRNA levels. The authors suggested that when solely mt-LSU is depleted, mt-SSU is available and capable of recruiting mature mt-mRNAs from the LRPPRC/SLIRP complex, thus depriving them of protection from degradation. Under normal conditions, the formation of the ribosome stabilizes mt-mRNAs and protects them from degradation [111].

The decay of mtRNAs is mediated by a complex of polynucleotide phosphorylase (PNPase) and adenosine triphosphate (ATP)-dependent RNA helicase SUV3, referred to as the mitochondrial degradosome [112]. PNPase has 3′-5′ exoribonucleolytic activity and localizes to the intermembrane space and mitochondrial matrix [112,113]. In the latter localization, PNPase interacts with SUV3, forming D-foci, where mtRNA degradation occurs (Figure 2) [112]. The mitochondrial degradosome is mainly involved in the degradation of mitochondrial antisense transcripts, with some contribution to the decay of mt-mRNAs [112,114]. The knockdown of either SUV3 or PNPase results in the strong accumulation of antisense transcripts, the appearance of mtRNAs with extended poly(A)-tails as well as degradation intermediates [56,112].

The RNA surveillance function of the degradosome is essential to maintain mitochondrial gene expression. It has been shown that dysfunction of the degradosome results in the accumulation of R-loops in mtDNA, leading to the instability of the mitochondrial genome [115]. Similarly, by controlling the levels of mitochondrial antisense transcripts, the degradosome regulates the level of mitochondrial double-stranded RNA (mt-dsRNA). A strong upregulation of mt-dsRNA was observed upon SUV3 or PNPase silencing [116]. Notably, PNPase has a diverse function in controlling mt-dsRNA, depending on the location within mitochondria [116]. As a subunit of the degradosome complex, PNPase counteracts the accumulation of dsRNA in the mitochondrial matrix. This function depends on SUV3 helicase activity [116]. On the other hand, a fraction of PNPase that localizes to the intermembrane space, where SUV3 is absent [117], prevents the release of mt-dsRNA into the cytosol, precluding the sterile inflammation [116].

The final products of PNPase/SUV3-mediated mtRNA degradation are short oligonucleotides that are removed by RNA exonuclease 2 (REXO2) [118]. REXO2 has 3′ to 5′ oligonuclease activity toward short single-stranded RNAs and DNAs, and its silencing results in the accumulation of short RNA species and double-stranded RNAs [119]. Furthermore, REXO2 knockout results in the disruption of mitochondrial protein synthesis [118]. REXO2 activity in mitochondria is considered to enable the recycling of monoribonucleotides and prevent the accumulation of short oligoribonucleotides that could affect degradosome function and stimulate promoter-independent transcription [119,120,121].

Another RBP that is engaged in mtRNA processing is GRSF1, which belongs to the quasi-RRM protein family [122]. The protein exists in human cells in two isoforms, one of which is targeted to MRGs where it co-localizes with nascent mtRNAs and RNase P [13]. The second isoform localizes to the nucleus and cytosol [13]. The loss of GRSF1 results in a slight increase in unprocessed transcripts and is required for mitoribosome assembly [13,123]. Recent studies show that GRSF1 associates with the PNPase/SUV3 complex, thus indicating its involvement in mtRNA decay [124]. A comprehensive RNA-sequencing analysis revealed that non-coding, antisense mtRNAs that are prone to form G-quadruplex structures (G4) accumulate upon degradosome or GRSF1 dysfunction. Further, biochemical and biophysical experiments confirmed that GRSF1/degradosome substrates form G4s. It was found that the binding of G4s by GRSF1 melts these structure, which in turn facilitates their degradation by the degradosome [124]. Interestingly, phylogenetic analysis revealed that GRSF1 proteins are distinct from other quasi-RRMs and that they appeared relatively late in evolution, namely in vertebrates, which have G4-rich mitochondrial genomes [124]. Therefore, the appearance of GRSF1 in mitochondria is likely to be an evolutionary adaptation that occurred when mitochondrial genomes underwent a transition from G4-poor to G4-rich, which enabled the control of the levels of RNAs that form G-quadruplexes.

## 5. Unresolved Issues

Recent years have brought discoveries of numerous proteins involved in post-transcriptional mtRNA processing, indicating that the regulation of mitochondrial gene expression that results in the synthesis of merely 13 OXPHOS proteins has unexpected complexity. Multiple studies revealed that mutations within the nuclear-encoded enzymes involved in mtRNA metabolism affect mtRNA maturation and may lead to human mitochondrial diseases [125]. Nevertheless, many aspects of mtRNA metabolism and the pathogenesis of mitochondrial syndromes remain unsolved. In this section, we highlight current issues and hypotheses that remain poorly understood and require further investigation.

### 5.1. Processing of Non-Canonical Sites in Primary Polycistronic Transcripts

The majority of mitochondrial mRNA and rRNA sequences are flanked by tRNAs. Hence, they are released from primary polycistronic transcripts along with tRNA processing by RNase P and ELAC2. However, there are also non-canonical junctions, where no tRNAs are present and where the transcripts are nonetheless released. For example, bicistronic *MT-ATP8*/*6* mRNA can be identified as a stable precursor transcript that contains the adjacent *MT-CO3* mRNA [35]. The junction of *MT-ATP8*/*6* mRNA and *MT-CO3* mRNA, where no tRNA is present, is likely not to be processed as efficiently as other cleavage sites, resulting in an increase in the stability and steady-state levels of this precursor. Another peculiarity of *MT-CO3* transcript processing was recently discovered. Based on the circularized RNA sequencing of RNAs from mouse heart mitochondria and a 5′-phosphate-dependent exonuclease digestion assay, *MT-CO3* mRNA was suggested to be lacking a 5′ monophosphate in contrast to other mt-mRNAs [126]. As mentioned above, FASTKD5 [7] and its interactor NLRX1 [45] may be involved in the processing of the *MT-ATP8*/*6-CO3* transcript and other non-canonical junctions. Another non-canonical processing site between *MT-ND5* and *MT-CYB* may be processed with the involvement of the FASTKD4 protein. Upon FASTKD4 depletion, the *MT-ND5-CYB* precursor accumulates, accompanied by a decrease in some mature transcript levels, including *MT-ND5* and *MT-CYB* [39]. To date, it has not been clarified as to whether FASTKD4 and FASTKD5 have endonuclease activity. Thus, whether the processing of these transcripts requires additional factors remains unknown.

### 5.2. Post-Transcriptional RNA Uridylation in Human Mitochondria

The untemplated addition of uridines at the 3′ end of RNA, named RNA uridylation, is a post-transcriptional modification of nuclear coding and non-coding RNAs, spread widely among different species, including fungi, trypanosomes, plants, and animals [127,128]. Notably, RNA uridylation was also observed in mitochondrial systems. In the mitochondria of *Trypanosoma brucei*, aberrant 12*S* rRNAs are marked for degradation by the addition of oligo(U) tails [129]. Similarly, the 3′-end uridylation of *T. brucei* mt-mRNAs stimulates their decay [130]. The phenomenon of RNA uridylation has also been observed in human mitochondria, but is still far from understood. Some improperly processed mtRNAs were reported to bear oligo- or poly(U) tails [56,131]. It is tempting to speculate that the addition of poly(U) or oligo(U) tails marks such abnormal RNAs and directs them for degradation. However, this hypothesis requires experimental validation.

### 5.3. Mitochondrial RNA Editing

Among post-transcriptional RNA modifications, one can distinguish RNA editing, which causes the transcripts to have a different sequence to that of the DNA template. RNA editing refers to site-specific alteration that may involve the deletion, insertion, or base substitution of nucleotides within an RNA molecule, though excluding RNA splicing and polyadenylation. This phenomenon has been observed in the mitochondrial-, chloroplast-, and nuclear-encoded RNAs of most eukaryotic organisms. For example, in humans, nuclear RNA editing occurs in the primate-specific transposable Alu elements that form dsRNA structures [132]. Using ultra-deep RNA-sequencing, Bazak et al. confirmed that virtually all adenosines within dsRNA-forming Alu sequences are subjected to adenosine-to-inosine transition [132]. During translation, inosine is recognized as guanosine, and thus, nucleotide conversion may change the amino acid sequence [132].

RNA editing in mitochondria is observed among numerous eukaryotic organisms, and it has been suggested that mtRNA editing leads to the restoration of ancestral protein sequences [133] (and references therein). This seems to be also relevant to human mitochondrial genetic systems, at least to some extent. Bar-Yaacov et al., using deep sequencing of human mtDNA and mtRNA, identified three sites harboring RNA–DNA differences (RDDs). Among them, A-to-U or A-to-G RDD at position 2617 located within the 16*S* rRNA has been identified in humans and non-human primates. RDD at this position has been suggested to restore the ancestral form of 16*S* rRNA and stabilize the mt-LSU structure [134]. The role of two other RDDs occurring at position 295 within the non-coding D-loop and at position 13710, a third codon position of alanine in the *MT-ND5*, remains unclear. Theories about the functional importance of the RDDs in human mitochondria and the mitochondrial RNA editing enzymes remain questionable and require further investigation.

### 5.4. Links between mtDNA Transcription and Mitoribosome Assembly

Mitochondrial ribosomes are built from components that are encoded in two genomes: protein components that are encoded in the nuclear genome, and RNA components that are encoded in the mitochondrial genome. Therefore, the biogenesis of the mitoribosome requires the coordination of rRNA transcription by mitochondrial transcription machinery and the synthesis of mitoribosome proteins in the cytoplasm and their import into mitochondria. The precise mechanisms that coordinate mtDNA transcription and ribosome assembly in mitochondria are unknown. However, there are some indications of the proteins that may connect these processes. MRPL12 protein, a component of the mt-LSU that also occurs as a “free pool”, has already been mentioned. This “free pool” stimulates mitochondrial transcription, suggesting that MRPL12 can coordinate the rRNA synthesis rate with the accessibility of the mitoribosomal proteins [25]. Other protein components of the mitoribosome have also been identified in the form of a stable mitoribosome-unbound state [25,135], indicating that some mitoribosomal proteins may have additional functions. MTRES1 is another mitochondrial protein that could couple mitochondrial transcription and translation processes. MTRES1 was shown to interact with mitochondrial transcription machinery [26] and associate with the mitoribosomes participating in translation quality control [136]. Another example of a protein that connects mitochondrial translation and transcription is the methyltransferase MRM3. This 16*S* rRNA-modifying enzyme has been shown to interact with the mt-LSU and with non-ribosomal proteins that may be engaged in coordinating rRNA transcription and mitoribosome assembly [91]. Notably, partial assembly of the mitoribosome large subunit has been suggested to occur co-transcriptionally [135]. It was observed that the disruption of mtRNA processing by MRPP3 knockout results in a decrease in mature 12*S* and 16*S* rRNA transcripts and the accumulation of an unprocessed precursor, including both 12*S* and 16*S* rRNA. Nevertheless, the mt-LSU can still assemble [135]. Simultaneously, the upregulation of mtDNA transcription was observed [135], suggesting crosstalk between the processing of primary transcripts, RNA synthesis, and mitoribosome biogenesis. A molecular mechanism that leads to such an interplay remains to be discovered.

### 5.5. Regulation of Mitochondrial Gene Expression

Most RNAs encoded by the same strand of mtDNA are synthetized with the same frequency. Nevertheless, the levels of specific mature mitochondrial transcripts differ significantly [46,137], indicating that the post-transcriptional processing of mtRNAs plays an important role in the regulation of mitochondrial gene expression. These processes include precursor cleavage, mtRNA modifications, stabilization, and decay, all of which occur in RNA granules [13,123,138] or D-foci [112]; submitochondrial compartments that are enriched in RBPs. To date, the mechanisms that lead to differential steady-state transcript levels have not been revealed.

Mitochondrial translation could account for a central layer of controlling of mitochondrial gene expression. This level of gene expression regulation plays an important role in bacteria, where multiple genes are transcribed as a single polycistronic transcript [139]. For mammalian cytoplasmic ribosomes, specific ribosomal proteins can promote the translation of specific pools of transcripts [140,141]. Likewise, in the yeast *Saccharomyces cerevisiae*, the translation of mitochondrial transcripts is mediated by specific translational activators that recognize sequences in 5′ UTRs of mRNAs [142]. In contrast to transcripts that are encoded in the cell nucleus and yeast mitochondrial genome, mammalian mtDNA-encoded transcripts either lack UTRs or possess UTRs that are only several nucleotides long [60]. Nevertheless, there are indications that mitochondrial translation may be regulated in a transcript-specific manner. A specific *MT-CO1* mRNA regulator has been identified. A translational activator of cytochrome c oxidase 1 (TACO1) interacts with *MT-CO1* mRNA and the mitochondrial ribosome. The loss of TACO1 specifically disrupts the translation of *MT-CO1* in mammalian mitochondria [143,144]. It remains to be seen whether the translation of other mt-mRNA is regulated by specific RBPs.

An emerging aspect of mitochondrial biology in human cells is the role of mt-dsRNA in mitochondrial gene expression and cell homeostasis. So far, the functional importance of the mt-dsRNA species has been revealed under abnormal conditions when dysfunction of the mtRNA surveillance pathway results in the accumulation of mt-dsRNAs and their escape to the cytoplasm, where they trigger a sterile inflammation response [145]. Nothing is known about the role of mt-dsRNA under physiological conditions. Nevertheless, it has been suggested that microRNAs are present in the mitochondrial transcriptome [146,147]. Moreover, some components of RNAi machinery, such as Ago2, were reported to be active in human mitochondria [148]. Thus, it is likely that RNAi-like mechanisms can operate in human mitochondria. If so, this would be an important mechanism of gene-specific regulation. However, the mitochondrial localization and function of RNAi machinery elements have not been studied systematically, with the exception of a report that shows a surprising ability to transfect mitochondria [149]. In summary, further study on the interesting concept of RNAi-mediated regulation of human mitochondrial gene expression is required.

## 6. Conclusions

RNA metabolism in human mitochondria is a complex process that requires both the spatial and temporal coordination of different mechanisms. This coordination occurs in the mitochondria, which is at least partially facilitated by the existence of RNA granules, and in the nucleocytoplasmic compartment, in which proteins that are encoded in the nuclear genome are involved in the regulation of mtRNA metabolism. We are only just beginning to understand how this coordination works. An important goal for future research is to unravel the intricacies of mtRNA metabolism regulation.

## Abbreviations

aARS	aminoacyl-tRNA synthetases
ATP6	ATP synthase membrane subunit 6
ATP8	ATP synthase membrane subunit 8
CO1	cytochrome c oxidase subunit 1
CO2	cytochrome c oxidase subunit 2
CO3	cytochrome c oxidase subunit 3
CSBII	G-rich sequence block II
CYB	cytochrome b
ELAC2	zinc phosphodiesterase ELAC protein 2
FASTK	Fas-activated serine/threonine kinase
G4	G-quadruplex
GRSF1	G-rich sequence factor 1
HSD17B10	hydroxysteroid 17-b dehydrogenase 10
HSP	H-strand promoter
LRPPRC	leucine-rich pentatricopeptide repeat-containing protein
LSP	L-strand promoter
MRG	mitochondrial RNA granules
MRPL12	mitochondrial ribosomal protein L7/L12
MRPs	mitoribosomal proteins
MTERF1	mitochondrial transcription termination factor 1
mt-LSU	mitochondrial ribosome large subunit
MTPAP	mitochondrial poly(A) polymerase
MTRES1	mitochondrial transcription rescue factor 1
MTS	mitochondrial targeting sequence
mtSSB (SSBP1)	single-stranded DNA-binding protein
mt-SSU	mitochondrial ribosome small subunit
NCR	non-coding regulatory region
ND1	NADH-ubiquinone oxidoreductase chain 1
ND2	NADH-ubiquinone oxidoreductase chain 2
ND3	NADH-ubiquinone oxidoreductase chain 3
ND4	NADH-ubiquinone oxidoreductase chain 4
ND4L	NADH-ubiquinone oxidoreductase chain 4L
ND5	NADH-ubiquinone oxidoreductase chain 5
ND6	NADH-ubiquinone oxidoreductase chain 6
OXPHOS	oxidative phosphorylation system
PDE12	3′ exonuclease phosphodiesterase 12
PNPase (PNPT1)	polynucleotide phosphorylase
POLG	polymerase γ
POLRMT	mitochondrial RNA polymerase
PPR	pentatricopeptide repeat
PRORP	protein-only RNase P catalytic subunit
RAP	RNA-binding domain abundant in apicomplexans
RBP	RNA-binding protein
REXO2	RNA exonuclease 2
RNase P	endoribonuclease P
RRM	RNA recognition motif
SIRT1	protein deacetylase sirtuin-1
SLIRP	stem-loop-interacting RNA-binding protein
SUV3 (SUPV3L1)	ATP-dependent RNA helicase SUPV3L1
TEFM	mitochondrial transcription elongation factor
TFAM	mitochondrial transcription factor A
TFB2M	mitochondrial transcription factor B2
TRMT10C	tRNA methyltransferase 10C

## References

[1] Robberson D.L., Clayton D.A. (1972). Replication of Mitochondrial DNA in Mouse L Cells and Their Thymidine Kinase- Derivatives: Displacement Replication on a Covalently-Closed Circular Template. Proc. Natl. Acad. Sci. USA.

[2] Anderson S., Bankier A.T., Barrell B.G., de Bruijn M.H.L., Coulson A.R., Drouin J., Eperon I.C., Nierlich D.P., Roe B.A., Sanger F. (1981). Sequence and organization of the human mitochondrial genome. Nature.

[3] Istiaq Alam T., Kanki T., Muta T., Ukaji K., Abe Y., Nakayama H., Takio K., Hamasaki N., Kang D. (2003). Human mitochondrial DNA is packaged with TFAM. Nucleic Acids Res..

[4] Garrido N., Griparic L., Jokitalo E., Wartiovaara J., van der Bliek A.M., Spelbrink J.N. (2003). Composition and dynamics of human mitochondrial nucleoids. Mol. Biol. Cell.

[5] Bogenhagen D.F., Wang Y., Shen E.L., Kobayashi R. (2003). Protein components of mitochondrial DNA nucleoids in higher eukaryotes. Mol. Cell. Proteom..

[6] Bogenhagen D.F. (2012). Mitochondrial DNA nucleoid structure. Biochim. Biophys. Acta.

[7] Antonicka H., Shoubridge E.A. (2015). Mitochondrial RNA Granules Are Centers for Posttranscriptional RNA Processing and Ribosome Biogenesis. Cell Rep..

[8] Jourdain A.A., Boehm E., Maundrell K., Martinou J.-C. (2016). Mitochondrial RNA granules: Compartmentalizing mitochondrial gene expression. J. Cell Biol..

[9] Hensen F., Potter A., van Esveld S.L., Tarrés-Solé A., Chakraborty A., Solà M., Spelbrink J.N. (2019). Mitochondrial RNA granules are critically dependent on mtDNA replication factors Twinkle and mtSSB. Nucleic Acids Res..

[10] Kaufman B.A., Durisic N., Mativetsky J.M., Costantino S., Hancock M.A., Grutter P., Shoubridge E.A. (2007). The Mitochondrial Transcription Factor TFAM Coordinates the Assembly of Multiple DNA Molecules into Nucleoid-like Structures. Mol. Biol. Cell.

[11] Kukat C., Davies K.M., Wurm C.A., Spåhr H., Bonekamp N.A., Kühl I., Joos F., Polosa P.L., Park C.B., Posse V. (2015). Cross-strand binding of TFAM to a single mtDNA molecule forms the mitochondrial nucleoid. Proc. Natl. Acad. Sci. USA.

[12] Ježek P., Špaček T., Tauber J., Pavluch V. (2019). Mitochondrial Nucleoids: Superresolution microscopy analysis. Int. J. Biochem. Cell Biol..

[13] Jourdain A.A., Koppen M., Wydro M., Rodley C.D., Lightowlers R.N., Chrzanowska-Lightowlers Z.M., Martinou J.-C. (2013). GRSF1 Regulates RNA Processing in Mitochondrial RNA Granules. Cell Metab..

[14] Tu Y.-T., Barrientos A. (2015). The Human Mitochondrial DEAD-Box Protein DDX28 Resides in RNA Granules and Functions in Mitoribosome Assembly. Cell Rep..

[15] Litonin D., Sologub M., Shi Y., Savkina M., Anikin M., Falkenberg M., Gustafsson C.M., Temiakov D. (2010). Human mitochondrial transcription revisited: Only TFAM and TFB2M are required for transcription of the mitochondrial genes in vitro. J. Biol. Chem..

[16] Minczuk M., He J., Duch A.M., Ettema T.J., Chlebowski A., Dzionek K., Nijtmans L.G.J., Huynen M.A., Holt I.J. (2011). TEFM (c17orf42) is necessary for transcription of human mtDNA. Nucleic Acids Res..

[17] Hillen H.S., Temiakov D., Cramer P. (2018). Structural basis of mitochondrial transcription. Nat. Struct. Mol. Biol..

[18] Hillen H.S., Morozov Y.I., Sarfallah A., Temiakov D., Cramer P. (2017). Structural Basis of Mitochondrial Transcription Initiation. Cell.

[19] Yakubovskaya E., Guja K.E., Eng E.T., Choi W.S., Mejia E., Beglov D., Lukin M., Kozakov D., Garcia-Diaz M. (2014). Organization of the human mitochondrial transcription initiation complex. Nucleic Acids Res..

[20] Morozov Y.I., Parshin A.V., Agaronyan K., Cheung A.C.M., Anikin M., Cramer P., Temiakov D. (2015). A model for transcription initiation in human mitochondria. Nucleic Acids Res..

[21] Ringel R., Sologub M., Morozov Y.I., Litonin D., Cramer P., Temiakov D. (2011). Structure of human mitochondrial RNA polymerase. Nature.

[22] Cermakian N., Ikeda T.M., Miramontes P., Lang B.F., Gray M.W., Cedergren R. (1997). On the evolution of the single-subunit RNA polymerases. J. Mol. Evol..

[23] Posse V., Gustafsson C.M. (2017). Human Mitochondrial Transcription Factor B2 Is Required for Promoter Melting during Initiation of Transcription. J. Biol. Chem..

[24] Wang Z., Cotney J., Shadel G.S. (2007). Human mitochondrial ribosomal protein MRPL12 interacts directly with mitochondrial RNA polymerase to modulate mitochondrial gene expression. J. Biol. Chem..

[25] Surovtseva Y.V., Shutt T.E., Cotney J., Cimen H., Chen S.Y., Koc E.C., Shadel G.S. (2011). Mitochondrial Ribosomal Protein L12 selectively associates with human mitochondrial RNA polymerase to activate transcription. Proc. Natl. Acad. Sci. USA.

[26] Kotrys A.V., Cysewski D., Czarnomska S.D., Pietras Z., Borowski L.S., Dziembowski A., Szczesny R.J. (2019). Quantitative proteomics revealed C6orf203/MTRES1 as a factor preventing stress-induced transcription deficiency in human mitochondria. Nucleic Acids Res..

[27] Agaronyan K., Morozov Y.I., Anikin M., Temiakov D. (2015). Mitochondrial biology. Replication-transcription switch in human mitochondria. Science.

[28] Chang D.D., Clayton D.A. (1985). Priming of human mitochondrial DNA replication occurs at the light-strand promoter. Proc. Natl. Acad. Sci. USA.

[29] Wanrooij P.H., Uhler J.P., Simonsson T., Falkenberg M., Gustafsson C.M. (2010). G-quadruplex structures in RNA stimulate mitochondrial transcription termination and primer formation. Proc. Natl. Acad. Sci. USA.

[30] Wanrooij P.H., Uhler J.P., Shi Y., Westerlund F., Falkenberg M., Gustafsson C.M. (2012). A hybrid G-quadruplex structure formed between RNA and DNA explains the extraordinary stability of the mitochondrial R-loop. Nucleic Acids Res..

[31] Pham X.H., Farge G., Shi Y., Gaspari M., Gustafsson C.M., Falkenberg M. (2006). Conserved sequence box II directs transcription termination and primer formation in mitochondria. J. Biol. Chem..

[32] Terzioglu M., Ruzzenente B., Harmel J., Mourier A., Jemt E., López M.D., Kukat C., Stewart J.B., Wibom R., Meharg C. (2013). MTERF1 binds mtDNA to prevent transcriptional interference at the light-strand promoter but is dispensable for rRNA gene transcription regulation. Cell Metab..

[33] Ojala D., Montoya J., Attardi G. (1981). tRNA punctuation model of RNA processing in human mitochondria. Nature.

[34] Holzmann J., Frank P., Löffler E., Bennett K.L., Gerner C., Rossmanith W. (2008). RNase P without RNA: Identification and Functional Reconstitution of the Human Mitochondrial tRNA Processing Enzyme. Cell.

[35] Brzezniak L.K., Bijata M., Szczesny R.J., Stepien P.P. (2011). Involvement of human ELAC2 gene product in 3’ end processing of mitochondrial tRNAs. RNA Biol..

[36] Jarrous N., Reiner R., Wesolowski D., Mann H., Guerrier-Takada C., Altman S. (2001). Function and subnuclear distribution of Rpp21, a protein subunit of the human ribonucleoprotein ribonuclease P. RNA.

[37] Rossmanith W. (2011). Localization of Human RNase Z Isoforms: Dual Nuclear/Mitochondrial Targeting of the ELAC2 Gene Product by Alternative Translation Initiation. PLoS ONE.

[38] Jourdain A.A., Popow J., de la Fuente M.A., Martinou J.-C., Anderson P., Simarro M. (2017). The FASTK family of proteins: Emerging regulators of mitochondrial RNA biology. Nucleic Acids Res..

[39] Boehm E., Zaganelli S., Maundrell K., Jourdain A.A., Thore S., Martinou J.-C. (2017). FASTKD1 and FASTKD4 have opposite effects on expression of specific mitochondrial RNAs, depending upon their endonuclease-like RAP domain. Nucleic Acids Res..

[40] Jourdain A.A., Koppen M., Rodley C.D., Maundrell K., Gueguen N., Reynier P., Guaras A.M., Enriquez J.A., Anderson P., Simarro M. (2015). A Mitochondria-Specific Isoform of FASTK Is Present In Mitochondrial RNA Granules and Regulates Gene Expression and Function. Cell Rep..

[41] Popow J., Alleaume A.-M., Curk T., Schwarzl T., Sauer S., Hentze M.W. (2015). FASTKD2 is an RNA-binding protein required for mitochondrial RNA processing and translation. RNA.

[42] Ghezzi D., Saada A., D’Adamo P., Fernandez-Vizarra E., Gasparini P., Tiranti V., Elpeleg O., Zeviani M. (2008). FASTKD2 Nonsense Mutation in an Infantile Mitochondrial Encephalomyopathy Associated with Cytochrome C Oxidase Deficiency. Am. J. Hum. Genet..

[43] Yoo D.H., Choi Y.-C., Nam D.E., Choi S.S., Kim J.W., Choi B.-O., Chung K.W. (2017). Identification of FASTKD2 compound heterozygous mutations as the underlying cause of autosomal recessive MELAS-like syndrome. Mitochondrion.

[44] Boehm E., Zornoza M., Jourdain A.A., Delmiro Magdalena A., García-Consuegra I., Torres Merino R., Orduña A., Martín M.A., Martinou J.-C., De la Fuente M.A. (2016). Role of FAST Kinase Domains 3 (FASTKD3) in Post-transcriptional Regulation of Mitochondrial Gene Expression. J. Biol. Chem..

[45] Singh K., Sripada L., Lipatova A., Roy M., Prajapati P., Gohel D., Bhatelia K., Chumakov P.M., Singh R. (2018). NLRX1 resides in mitochondrial RNA granules and regulates mitochondrial RNA processing and bioenergetic adaptation. Biochim. Biophys. Acta BBA Mol. Cell Res..

[46] Mercer T.R., Neph S., Dinger M.E., Crawford J., Smith M.A., Shearwood A.-M.J., Haugen E., Bracken C.P., Rackham O., Stamatoyannopoulos J.A. (2011). The human mitochondrial transcriptome. Cell.

[47] Tomecki R., Dmochowska A., Gewartowski K., Dziembowski A., Stepien P.P. (2004). Identification of a novel human nuclear-encoded mitochondrial poly(A) polymerase. Nucleic Acids Res..

[48] Nagaike T., Suzuki T., Katoh T., Ueda T. (2005). Human Mitochondrial mRNAs Are Stabilized with Polyadenylation Regulated by Mitochondria-specific Poly(A) Polymerase and Polynucleotide Phosphorylase. J. Biol. Chem..

[49] Wilson W.C., Hornig-Do H.-T., Bruni F., Chang J.H., Jourdain A.A., Martinou J.-C., Falkenberg M., Spåhr H., Larsson N.-G., Lewis R.J. (2014). A human mitochondrial poly(A) polymerase mutation reveals the complexities of post-transcriptional mitochondrial gene expression. Hum. Mol. Genet..

[50] Chang J.H., Tong L. (2012). Mitochondrial poly(A) polymerase and polyadenylation. Biochim. Biophys. Acta.

[51] Fiedler M., Rossmanith W., Wahle E., Rammelt C. (2015). Mitochondrial poly(A) polymerase is involved in tRNA repair. Nucleic Acids Res..

[52] Wende S., Bonin S., Götze O., Betat H., Mörl M. (2015). The identity of the discriminator base has an impact on CCA addition. Nucleic Acids Res..

[53] Pearce S.F., Rorbach J., Haute L.V., D’Souza A.R., Rebelo-Guiomar P., Powell C.A., Brierley I., Firth A.E., Minczuk M. (2021). Maturation of selected human mitochondrial tRNAs requires deadenylation. eLife.

[54] Toompuu M., Tuomela T., Laine P., Paulin L., Dufour E., Jacobs H.T. (2018). Polyadenylation and degradation of structurally abnormal mitochondrial tRNAs in human cells. Nucleic Acids Res..

[55] Slomovic S., Laufer D., Geiger D., Schuster G. (2005). Polyadenylation and Degradation of Human Mitochondrial RNA: The Prokaryotic Past Leaves Its Mark. Mol. Cell. Biol..

[56] Szczesny R.J., Borowski L.S., Brzezniak L.K., Dmochowska A., Gewartowski K., Bartnik E., Stepien P.P. (2010). Human mitochondrial RNA turnover caught in flagranti: Involvement of hSuv3p helicase in RNA surveillance. Nucleic Acids Res..

[57] Hajnsdorf E., Kaberdin V.R. (2018). RNA polyadenylation and its consequences in prokaryotes. Philos. Trans. R. Soc. B Biol. Sci..

[58] Tudek A., Lloret-Llinares M., Jensen T.H. (2018). The multitasking polyA tail: Nuclear RNA maturation, degradation and export. Philos. Trans. R. Soc. B Biol. Sci..

[59] Bratic A., Clemente P., Calvo-Garrido J., Maffezzini C., Felser A., Wibom R., Wedell A., Freyer C., Wredenberg A. (2016). Mitochondrial Polyadenylation Is a One-Step Process Required for mRNA Integrity and tRNA Maturation. PLoS Genet..

[60] Temperley R.J., Wydro M., Lightowlers R.N., Chrzanowska-Lightowlers Z.M. (2010). Human mitochondrial mRNAs—like members of all families, similar but different. Biochim. Biophys. Acta.

[61] Wydro M., Bobrowicz A., Temperley R.J., Lightowlers R.N., Chrzanowska-Lightowlers Z.M. (2010). Targeting of the cytosolic poly(A) binding protein PABPC1 to mitochondria causes mitochondrial translation inhibition. Nucleic Acids Res..

[62] Mudge S.J., Williams J.H., Eyre H.J., Sutherland G.R., Cowan P.J., Power D.A. (1998). Complex organisation of the 5′-end of the human glycine tRNA synthetase gene. Gene.

[63] Tolkunova E., Park H., Xia J., King M.P., Davidson E. (2000). The human lysyl-tRNA synthetase gene encodes both the cytoplasmic and mitochondrial enzymes by means of an unusual alternative splicing of the primary transcript. J. Biol. Chem..

[64] Sissler M., González-Serrano L.E., Westhof E. (2017). Recent Advances in Mitochondrial Aminoacyl-tRNA Synthetases and Disease. Trends Mol. Med..

[65] Nagao A., Suzuki T., Katoh T., Sakaguchi Y., Suzuki T. (2009). Biogenesis of glutaminyl-mt tRNAGln in human mitochondria. Proc. Natl. Acad. Sci. USA.

[66] Safra M., Sas-Chen A., Nir R., Winkler R., Nachshon A., Bar-Yaacov D., Erlacher M., Rossmanith W., Stern-Ginossar N., Schwartz S. (2017). The m 1 A landscape on cytosolic and mitochondrial mRNA at single-base resolution. Nature.

[67] Li X., Xiong X., Zhang M., Wang K., Chen Y., Zhou J., Mao Y., Lv J., Yi D., Chen X.-W. (2017). Base-resolution mapping reveals distinct m1A methylome in nuclear- and mitochondrial-encoded transcripts. Mol. Cell.

[68] Antonicka H., Choquet K., Lin Z., Gingras A., Kleinman C.L., Shoubridge E.A. (2017). A pseudouridine synthase module is essential for mitochondrial protein synthesis and cell viability. EMBO Rep..

[69] Vilardo E., Nachbagauer C., Buzet A., Taschner A., Holzmann J., Rossmanith W. (2012). A subcomplex of human mitochondrial RNase P is a bifunctional methyltransferase—Extensive moonlighting in mitochondrial tRNA biogenesis. Nucleic Acids Res..

[70] Suzuki T., Yashiro Y., Kikuchi I., Ishigami Y., Saito H., Matsuzawa I., Okada S., Mito M., Iwasaki S., Ma D. (2020). Complete chemical structures of human mitochondrial tRNAs. Nat. Commun..

[71] Nakano S., Suzuki T., Kawarada L., Iwata H., Asano K., Suzuki T. (2016). NSUN3 methylase initiates 5-formylcytidine biogenesis in human mitochondrial tRNA Met. Nat. Chem. Biol..

[72] Kawarada L., Suzuki T., Ohira T., Hirata S., Miyauchi K., Suzuki T. (2017). ALKBH1 is an RNA dioxygenase responsible for cytoplasmic and mitochondrial tRNA modifications. Nucleic Acids Res..

[73] Haag S., Sloan K.E., Ranjan N., Warda A.S., Kretschmer J., Blessing C., Hübner B., Seikowski J., Dennerlein S., Rehling P. (2016). NSUN3 and ABH1 modify the wobble position of mt-tRNA Met to expand codon recognition in mitochondrial translation. EMBO J..

[74] Umeda N., Suzuki T., Yukawa M., Ohya Y., Shindo H., Watanabe K., Suzuki T. (2005). Mitochondria-specific RNA-modifying Enzymes Responsible for the Biosynthesis of the Wobble Base in Mitochondrial tRNAs: Implications for the Molecular Pathogenesis of Human Mitochondrial Diseases. J. Biol. Chem..

[75] Boutoual R., Meseguer S., Villarroya M., Martín-Hernández E., Errami M., Martín M.A., Casado M., Armengod M.-E. (2018). Defects in the mitochondrial-tRNA modification enzymes MTO1 and GTPBP3 promote different metabolic reprogramming through a HIF-PPARγ-UCP2-AMPK axis. Sci. Rep..

[76] Sasarman F., Antonicka H., Horvath R., Shoubridge E.A. (2011). The 2-thiouridylase function of the human MTU1 (TRMU) enzyme is dispensable for mitochondrial translation. Hum. Mol. Genet..

[77] Yarham J.W., Lamichhane T.N., Pyle A., Mattijssen S., Baruffini E., Bruni F., Donnini C., Vassilev A., He L., Blakely E.L. (2014). Defective i6A37 Modification of Mitochondrial and Cytosolic tRNAs Results from Pathogenic Mutations in TRIT1 and Its Substrate tRNA. PLoS Genet..

[78] Powell C.A., Kopajtich R., D’Souza A.R., Rorbach J., Kremer L.S., Husain R.A., Dallabona C., Donnini C., Alston C.L., Griffin H. (2015). TRMT5 Mutations Cause a Defect in Post-transcriptional Modification of Mitochondrial tRNA Associated with Multiple Respiratory-Chain Deficiencies. Am. J. Hum. Genet..

[79] Reiter V., Matschkal D.M.S., Wagner M., Globisch D., Kneuttinger A.C., Müller M., Carell T. (2012). The CDK5 repressor CDK5RAP1 is a methylthiotransferase acting on nuclear and mitochondrial RNA. Nucleic Acids Res..

[80] Lin H., Miyauchi K., Harada T., Okita R., Takeshita E., Komaki H., Fujioka K., Yagasaki H., Goto Y., Yanaka K. (2018). CO2-sensitive tRNA modification associated with human mitochondrial disease. Nat. Commun..

[81] Zhou J.-B., Wang Y., Zeng Q.-Y., Meng S.-X., Wang E.-D., Zhou X.-L. (2020). Molecular basis for t6A modification in human mitochondria. Nucleic Acids Res..

[82] Van Haute L., Lee S.-Y., McCann B.J., Powell C.A., Bansal D., Vasiliauskaitė L., Garone C., Shin S., Kim J.-S., Frye M. (2019). NSUN2 introduces 5-methylcytosines in mammalian mitochondrial tRNAs. Nucleic Acids Res..

[83] Shinoda S., Kitagawa S., Nakagawa S., Wei F.-Y., Tomizawa K., Araki K., Araki M., Suzuki T., Suzuki T. (2019). Mammalian NSUN2 introduces 5-methylcytidines into mitochondrial tRNAs. Nucleic Acids Res..

[84] Laptev I., Shvetsova E., Levitskii S., Serebryakova M., Rubtsova M., Bogdanov A., Kamenski P., Sergiev P., Dontsova O. (2019). Mouse Trmt2B protein is a dual specific mitochondrial metyltransferase responsible for m5U formation in both tRNA and rRNA. RNA Biol..

[85] Chujo T., Suzuki T. (2012). Trmt61B is a methyltransferase responsible for 1-methyladenosine at position 58 of human mitochondrial tRNAs. RNA.

[86] Davis D.R. (1995). Stabilization of RNA stacking by pseudouridine. Nucleic Acids Res..

[87] Patton J.R., Bykhovskaya Y., Mengesha E., Bertolotto C., Fischel-Ghodsian N. (2005). Mitochondrial Myopathy and Sideroblastic Anemia (MLASA): Missense Mutation in the Pseudouridine Synthase 1 (PUS1) Gene Is Associated with the Loss of tRNA Pseudouridylation. J. Biol. Chem..

[88] Zaganelli S., Rebelo-Guiomar P., Maundrell K., Rozanska A., Pierredon S., Powell C.A., Jourdain A.A., Hulo N., Lightowlers R.N., Chrzanowska-Lightowlers Z.M. (2017). The Pseudouridine Synthase RPUSD4 Is an Essential Component of Mitochondrial RNA Granules. J. Biol. Chem..

[89] Nagaike T., Suzuki T., Tomari Y., Takemoto-Hori C., Negayama F., Watanabe K., Ueda T. (2001). Identification and Characterization of Mammalian Mitochondrial tRNA nucleotidyltransferases. J. Biol. Chem..

[90] Hyde S.J., Eckenroth B.E., Smith B.A., Eberley W.A., Heintz N.H., Jackman J.E., Doublié S. (2010). tRNAHis guanylyltransferase (THG1), a unique 3′-5′ nucleotidyl transferase, shares unexpected structural homology with canonical 5′-3′ DNA polymerases. Proc. Natl. Acad. Sci. USA.

[91] Lee K.-W., Okot-Kotber C., LaComb J.F., Bogenhagen D.F. (2013). Mitochondrial Ribosomal RNA (rRNA) Methyltransferase Family Members Are Positioned to Modify Nascent rRNA in Foci near the Mitochondrial DNA Nucleoid. J. Biol. Chem..

[92] Rorbach J., Boesch P., Gammage P.A., Nicholls T.J.J., Pearce S.F., Patel D., Hauser A., Perocchi F., Minczuk M. (2014). MRM2 and MRM3 are involved in biogenesis of the large subunit of the mitochondrial ribosome. Mol. Biol. Cell.

[93] Lee K.-W., Bogenhagen D.F. (2014). Assignment of 2′-O-Methyltransferases to Modification Sites on the Mammalian Mitochondrial Large Subunit 16 S Ribosomal RNA (rRNA). J. Biol. Chem..

[94] Bar-Yaacov D., Frumkin I., Yashiro Y., Chujo T., Ishigami Y., Chemla Y., Blumberg A., Schlesinger O., Bieri P., Greber B. (2016). Mitochondrial 16S rRNA Is Methylated by tRNA Methyltransferase TRMT61B in All Vertebrates. PLoS Biol..

[95] Seidel-Rogol B.L., McCulloch V., Shadel G.S. (2003). Human mitochondrial transcription factor B1 methylates ribosomal RNA at a conserved stem-loop. Nat. Genet..

[96] Metodiev M.D., Lesko N., Park C.B., Cámara Y., Shi Y., Wibom R., Hultenby K., Gustafsson C.M., Larsson N.-G. (2009). Methylation of 12S rRNA Is Necessary for In Vivo Stability of the Small Subunit of the Mammalian Mitochondrial Ribosome. Cell Metab..

[97] Liu X., Shen S., Wu P., Li F., Liu X., Wang C., Gong Q., Wu J., Yao X., Zhang H. (2019). Structural insights into dimethylation of 12S rRNA by TFB1M: Indispensable role in translation of mitochondrial genes and mitochondrial function. Nucleic Acids Res..

[98] Rozanska A., Richter-Dennerlein R., Rorbach J., Gao F., Lewis R.J., Chrzanowska-Lightowlers Z.M., Lightowlers R.N. (2017). The human RNA-binding protein RBFA promotes the maturation of the mitochondrial ribosome. Biochem. J..

[99] Metodiev M.D., Spåhr H., Loguercio Polosa P., Meharg C., Becker C., Altmueller J., Habermann B., Larsson N.-G., Ruzzenente B. (2014). NSUN4 Is a Dual Function Mitochondrial Protein Required for Both Methylation of 12S rRNA and Coordination of Mitoribosomal Assembly. PLoS Genet..

[100] Haute L.V., Hendrick A.G., D’Souza A.R., Powell C.A., Rebelo-Guiomar P., Harbour M.E., Ding S., Fearnley I.M., Andrews B., Minczuk M. (2019). METTL15 introduces N4-methylcytidine into human mitochondrial 12S rRNA and is required for mitoribosome biogenesis. Nucleic Acids Res..

[101] Chen H., Shi Z., Guo J., Chang K., Chen Q., Yao C., Haigis M.C., Shi Y. (2019). The human mitochondrial 12S rRNA m4C methyltransferase METTL15 is required for proper mitochondrial function. bioRxiv.

[102] Powell C.A., Minczuk M. (2020). TRMT2B is responsible for both tRNA and rRNA m5U-methylation in human mitochondria. RNA Biol..

[103] Suzuki T., Suzuki T. (2014). A complete landscape of post-transcriptional modifications in mammalian mitochondrial tRNAs. Nucleic Acids Res..

[104] Mukhopadhyay S., Deogharia M., Gupta R. (2021). Mammalian nuclear TRUB1, mitochondrial TRUB2, and cytoplasmic PUS10 produce conserved pseudouridine 55 in different sets of tRNA. RNA.

[105] Siira S.J., Spåhr H., Shearwood A.-M.J., Ruzzenente B., Larsson N.-G., Rackham O., Filipovska A. (2017). LRPPRC-mediated folding of the mitochondrial transcriptome. Nat. Commun..

[106] Ruzzenente B., Metodiev M.D., Wredenberg A., Bratic A., Park C.B., Cámara Y., Milenkovic D., Zickermann V., Wibom R., Hultenby K. (2012). LRPPRC is necessary for polyadenylation and coordination of translation of mitochondrial mRNAs. EMBO J..

[107] Lagouge M., Mourier A., Lee H.J., Spåhr H., Wai T., Kukat C., Silva Ramos E., Motori E., Busch J.D., Siira S. (2015). SLIRP Regulates the Rate of Mitochondrial Protein Synthesis and Protects LRPPRC from Degradation. PLoS Genet..

[108] Spåhr H., Rozanska A., Li X., Atanassov I., Lightowlers R.N., Chrzanowska-Lightowlers Z.M.A., Rackham O., Larsson N.-G. (2016). SLIRP stabilizes LRPPRC via an RRM–PPR protein interface. Nucleic Acids Res..

[109] Aibara S., Singh V., Modelska A., Amunts A. (2020). Structural basis of mitochondrial translation. eLife.

[110] Chujo T., Ohira T., Sakaguchi Y., Goshima N., Nomura N., Nagao A., Suzuki T. (2012). LRPPRC/SLIRP suppresses PNPase-mediated mRNA decay and promotes polyadenylation in human mitochondria. Nucleic Acids Res..

[111] Bruni F., Proctor-Kent Y., Lightowlers R.N., Chrzanowska-Lightowlers Z.M. (2021). Messenger RNA delivery to mitoribosomes—hints from a bacterial toxin. FEBS J..

[112] Borowski L.S., Dziembowski A., Hejnowicz M.S., Stepien P.P., Szczesny R.J. (2013). Human mitochondrial RNA decay mediated by PNPase–hSuv3 complex takes place in distinct foci. Nucleic Acids Res..

[113] Lin C.L., Wang Y.-T., Yang W.-Z., Hsiao Y.-Y., Yuan H.S. (2012). Crystal structure of human polynucleotide phosphorylase: Insights into its domain function in RNA binding and degradation. Nucleic Acids Res..

[114] Pietras Z., Wojcik M.A., Borowski L.S., Szewczyk M., Kulinski T.M., Cysewski D., Stepien P.P., Dziembowski A., Szczesny R.J. (2018). Controlling the mitochondrial antisense—Role of the SUV3-PNPase complex and its co-factor GRSF1 in mitochondrial RNA surveillance. Mol. Cell. Oncol..

[115] Silva S., Camino L.P., Aguilera A. (2018). Human mitochondrial degradosome prevents harmful mitochondrial R loops and mitochondrial genome instability. Proc. Natl. Acad. Sci. USA.

[116] Dhir A., Dhir S., Borowski L.S., Jimenez L., Teitell M., Rötig A., Crow Y.J., Rice G.I., Duffy D., Tamby C. (2018). Mitochondrial double-stranded RNA triggers antiviral signalling in humans. Nature.

[117] Minczuk M., Piwowarski J., Papworth M.A., Awiszus K., Schalinski S., Dziembowski A., Dmochowska A., Bartnik E., Tokatlidis K., Stepien P.P. (2002). Localisation of the human hSuv3p helicase in the mitochondrial matrix and its preferential unwinding of dsDNA. Nucleic Acids Res..

[118] Bruni F., Gramegna P., Oliveira J.M.A., Lightowlers R.N., Chrzanowska-Lightowlers Z.M.A. (2013). REXO2 Is an Oligoribonuclease Active in Human Mitochondria. PLoS ONE.

[119] Szewczyk M., Malik D., Borowski L.S., Czarnomska S.D., Kotrys A.V., Klosowska-Kosicka K., Nowotny M., Szczesny R.J. (2020). Human REXO2 controls short mitochondrial RNAs generated by mtRNA processing and decay machinery to prevent accumulation of double-stranded RNA. Nucleic Acids Res..

[120] Chu L.-Y., Agrawal S., Chen Y.-P., Yang W.-Z., Yuan H.S. (2019). Structural insights into nanoRNA degradation by human Rexo2. RNA.

[121] Nicholls T.J., Spåhr H., Jiang S., Siira S.J., Koolmeister C., Sharma S., Kauppila J.H.K., Jiang M., Kaever V., Rackham O. (2019). Dinucleotide Degradation by REXO2 Maintains Promoter Specificity in Mammalian Mitochondria. Mol. Cell.

[122] Dominguez C., Fisette J.-F., Chabot B., Allain F.H.-T. (2010). Structural basis of G-tract recognition and encaging by hnRNP F quasi-RRMs. Nat. Struct. Mol. Biol..

[123] Antonicka H., Sasarman F., Nishimura T., Paupe V., Shoubridge E.A. (2013). The Mitochondrial RNA-Binding Protein GRSF1 Localizes to RNA Granules and Is Required for Posttranscriptional Mitochondrial Gene Expression. Cell Metab..

[124] Pietras Z., Wojcik M.A., Borowski L.S., Szewczyk M., Kulinski T.M., Cysewski D., Stepien P.P., Dziembowski A., Szczesny R.J. (2018). Dedicated surveillance mechanism controls G-quadruplex forming non-coding RNAs in human mitochondria. Nat. Commun..

[125] Van Haute L., Pearce S.F., Powell C.A., D’Souza A.R., Nicholls T.J., Minczuk M. (2015). Mitochondrial transcript maturation and its disorders. J. Inherit. Metab. Dis..

[126] Kuznetsova I., Siira S.J., Shearwood A.-M.J., Ermer J.A., Filipovska A., Rackham O. (2017). Simultaneous processing and degradation of mitochondrial RNAs revealed by circularized RNA sequencing. Nucleic Acids Res..

[127] Malecki M., Viegas S.C., Carneiro T., Golik P., Dressaire C., Ferreira M.G., Arraiano C.M. (2013). The exoribonuclease Dis3L2 defines a novel eukaryotic RNA degradation pathway. EMBO J..

[128] Warkocki Z., Liudkovska V., Gewartowska O., Mroczek S., Dziembowski A. (2018). Terminal nucleotidyl transferases (TENTs) in mammalian RNA metabolism. Philos. Trans. R. Soc. B Biol. Sci..

[129] Mattiacio J.L., Read L.K. (2008). Roles for TbDSS-1 in RNA surveillance and decay of maturation by-products from the 12S rRNA locus. Nucleic Acids Res..

[130] Aphasizheva I., Aphasizhev R. (2010). RET1-Catalyzed Uridylylation Shapes the Mitochondrial Transcriptome in Trypanosoma brucei. Mol. Cell. Biol..

[131] Slomovic S., Schuster G. (2008). Stable PNPase RNAi silencing: Its effect on the processing and adenylation of human mitochondrial RNA. RNA.

[132] Bazak L., Haviv A., Barak M., Jacob-Hirsch J., Deng P., Zhang R., Isaacs F.J., Rechavi G., Li J.B., Eisenberg E. (2014). A-to-I RNA editing occurs at over a hundred million genomic sites, located in a majority of human genes. Genome Res..

[133] Sloan D.B. (2017). Nuclear and mitochondrial RNA editing systems have opposite effects on protein diversity. Biol. Lett..

[134] Bar-Yaacov D., Avital G., Levin L., Richards A.L., Hachen N., Rebolledo Jaramillo B., Nekrutenko A., Zarivach R., Mishmar D. (2013). RNA-DNA differences in human mitochondria restore ancestral form of 16S ribosomal RNA. Genome Res..

[135] Rackham O., Busch J.D., Matic S., Siira S.J., Kuznetsova I., Atanassov I., Ermer J.A., Shearwood A.-M.J., Richman T.R., Stewart J.B. (2016). Hierarchical RNA Processing Is Required for Mitochondrial Ribosome Assembly. Cell Rep..

[136] Desai N., Yang H., Chandrasekaran V., Kazi R., Minczuk M., Ramakrishnan V. (2020). Elongational Stalling Activates Mitoribosome-associated Quality Control. Science.

[137] Piechota J., Tomecki R., Gewartowski K., Szczesny R., Dmochowska A., Kudła M., Dybczyńska L., Stepien P.P., Bartnik E. (2006). Differential stability of mitochondrial mRNA in HeLa cells. Acta Biochim. Pol..

[138] Iborra F.J., Kimura H., Cook P.R. (2004). The functional organization of mitochondrial genomes in human cells. BMC Biol..

[139] Quax T.E.F., Wolf Y.I., Koehorst J.J., Wurtzel O., van der Oost R., Ran W., Blombach F., Makarova K.S., Brouns S.J.J., Forster A.C. (2013). Differential Translation Tunes Uneven Production of Operon-Encoded Proteins. Cell Rep..

[140] Xue S., Tian S., Fujii K., Kladwang W., Das R., Barna M. (2015). RNA regulons in Hox 5′UTRs confer ribosome specificity to gene regulation. Nature.

[141] Shi Z., Fujii K., Kovary K.M., Genuth N.R., Röst H.L., Teruel M.N., Barna M. (2017). Heterogeneous ribosomes preferentially translate distinct subpools of mRNAs genome-wide. Mol. Cell.

[142] Naithani S., Saracco S.A., Butler C.A., Fox T.D. (2002). Interactions among COX1, COX2, andCOX3 mRNA-specific Translational Activator Proteins on the Inner Surface of the Mitochondrial Inner Membrane ofSaccharomyces cerevisiae. Mol. Biol. Cell.

[143] Weraarpachai W., Antonicka H., Sasarman F., Seeger J., Schrank B., Kolesar J.E., Lochmüller H., Chevrette M., Kaufman B.A., Horvath R. (2009). Mutation in TACO1, encoding a translational activator of COX I, results in cytochrome c oxidase deficiency and late-onset Leigh syndrome. Nat. Genet..

[144] Richman T.R., Spåhr H., Ermer J.A., Davies S.M.K., Viola H.M., Bates K.A., Papadimitriou J., Hool L.C., Rodger J., Larsson N.-G. (2016). Loss of the RNA-binding protein TACO1 causes late-onset mitochondrial dysfunction in mice. Nat. Commun..

[145] Kotrys A.V., Szczesny R.J. (2019). Mitochondrial Gene Expression and Beyond-Novel Aspects of Cellular Physiology. Cells.

[146] Bandiera S., Rüberg S., Girard M., Cagnard N., Hanein S., Chrétien D., Munnich A., Lyonnet S., Henrion-Caude A. (2011). Nuclear outsourcing of RNA interference components to human mitochondria. PLoS ONE.

[147] Ro S., Ma H.-Y., Park C., Ortogero N., Song R., Hennig G.W., Zheng H., Lin Y.-M., Moro L., Hsieh J.-T. (2013). The mitochondrial genome encodes abundant small noncoding RNAs. Cell Res..

[148] Zhang X., Zuo X., Yang B., Li Z., Xue Y., Zhou Y., Huang J., Zhao X., Zhou J., Yan Y. (2014). MicroRNA directly enhances mitochondrial translation during muscle differentiation. Cell.

[149] Gao K., Cheng M., Zuo X., Lin J., Hoogewijs K., Murphy M.P., Fu X.-D., Zhang X. (2021). Active RNA interference in mitochondria. Cell Res..

